# Motivating patients in cardiac rehabilitation programs: A multicenter randomized controlled trial

**DOI:** 10.5195/ijt.2021.6365

**Published:** 2021-06-22

**Authors:** Helle Spindler, Malene Hollingdal, Jens Refsgaard, Birthe Dinesen

**Affiliations:** 1 Department of Psychology and Behavioral Sciences, Aarhus University, Denmark; 2 Cardiology Ward, Regional Hospital Viborg, Viborg, Denmark; 3 Laboratory for Welfare Technology - Telehealth & Telerehabilitation, Sport Sciences-Performance and Technology, Department of Health Science and Technology, Aalborg University, Denmark

**Keywords:** Heart failure, Motivation, Psychological distress, Telerehabilitation

## Abstract

Concerns have been raised about motivation and psychological distress when implementing telerehabilitation in patients with heart failure. The current study compared conventional and telerehabilitation in two groups (n=67; n=70) of patients with heart failure at 0, 6, and 12 months on measures of motivation (Self-Determination Theory measures) and psychological distress (Hospital Anxiety and Depression scale). We found no significant changes in motivation across groups, although our telerehabilitation group had a slightly lower level of controlled motivation and higher levels of relatedness. In addition, there were no differences between groups with regard to psychological distress. This study demonstrates that telerehabilitation motivates patients with heart failure to the same degree as conventional rehabilitation, and that telerehabilitation is not associated with increased psychological distress. As such, telerehabilitation offers an alternative to conventional rehabilitation and addresses some of the barriers for participating in rehabilitation identified in the literature.

Heart failure (HF) is one of the most common causes of hospitalization, re-hospitalizations, and mortality affecting about 26 million people globally (Cook et al., 2014; Savarese & Lund, 2017). An important part of the treatment and rehabilitation process in patients with HF is to provide education and support patients in independent disease management, i.e. self-care (Jaarsma et al., 2003). Evidence shows that increased self-care in patients with HF is associated with increased treatment compliance and quality of life (Buck et al., 2012; Toback & Clark, 2017), and reduced re-hospitalizations (Lee et al., 2011; McAlister et al., 2004). However, sociodemographic factors, long distance travelling to reach the rehabilitation site, or having to work during rehabilitation may reduce adherence to rehabilitation (Balady et al., 2011; Cranen et al., 2012; Dalleck et al., 2011; Piotrowicz et al., 2010; Piotrowicz, et al., 2016). Consequently, it may be important to support patient motivation for rehabilitation and disease management as key factors in secondary prevention.

Telerehabilitation (TR) is increasingly adopted as a means to overcome some of these barriers (Brouwers et al., 2020; Knudsen et al., 2019). TR entails the delivery of rehabilitation via information and communication technologies over distance (Brennan et al., 2010), and is used increasingly on a global scale, especially so during the COVID-19 pandemic. Although studies are few, they all indicate that TR is effective, either as a stand-alone strategy, or combined with conventional rehabilitation (CR) (Anderson et al, 2017; Brouwers et al., 2020; Frederix et al., 2015; Spindler et al., 2019). A recent review suggests that TR for patients with HF is well-accepted, safe, effective, and with high adherence (Piotrowicz, 2017).

The main goals of HF rehabilitation, regardless of the mode of delivery, is to provide education and support for patients when making lifestyle changes and implementing self-care in everyday life. It is, however, important to consider the impact of patient motivation and psychological distress (i.e., anxiety and depression), since low motivation and psychological distress have been associated with decreased self-care (Jarsmaa et al., 2017). Psychological distress is prevalent in patients with HF (Scherer et al., 2007; Yohannes et al., 2010) and has been associated with decreased self-care in patients with HF (Jaarsma et al., 2017), as well as decreased motivation in patients with diabetes (Egede & Osborn, 2010). This association may reflect that psychological distress, such as depression, negatively affects adherence to treatment. However, results regarding depression and adherence in patients with HF are somewhat conflicting and differ depending on the type of adherence examined (Kessing et al., 2016; Schweitzer et al., 2007; Shen & Maeda., 2018; Tang et al., 2014).

In our earlier work (the Teledi@log project, Spindler et al., 2019), we adopted the Self-Determination Theory (SDT) conceptualization of motivation as a theoretical background in our design process. This decision was in accordance with a recent review stressing that using theory to inform the design process may increase the effectiveness of the intervention when developing rehabilitation programs for behavior change (Cho et al., 2018). The Teledi@log project found that patient motivation was comparable across TR and CR with regard to lifestyle changes and self-care. Therefore, we also used the SDT to inform our design process in the current study.

Previous studies have used the SDT in relation to motivation for physical exercise in CR with promising results (Rahman et al., 2015; Sweet et al., 2011). The SDT posits two types of motivation: intrinsic and extrinsic motivation. Intrinsic motivation is characterized by desire and interest as the key motivational factors. In contrast, a patient driven by extrinsic motivation attempts to avoid punishment or obtain rewards. The aim of rehabilitation is to promote intrinsic motivation as this enables individuals to maintain their health behavior over time (Ryan et al., 2008). The SDT argues that promoting intrinsic motivation requires three key aspects (1) autonomy: when a person considers their health behavior to be in accordance with their own values and beliefs, (2) competency: when a person feels that they possess the knowledge and skills to perform this health behavior (i.e., a term closely related to self-efficacy), and (3) relatedness: when a person feels supported by others. As such, the SDT provides a theoretical background with an emerging evidence base for understanding the nature of the individual's motivation. SDT may be highly relevant with respect to creating the individually tailored solutions within the new health care paradigm of personalized medicine (Dinesen et al., 2016).

Based on these insights, we used participatory design (Clemensen et al., 2017) to develop a TR program for patients with HF called the Future Patient Telerehabilitation (FPT) program (Joensson et al., 2019). We invited patients with HF, their partners, health care professional, and researchers to participate in the design process, while the process was also informed by both research and SDT.

The aim of this psychological substudy was to evaluate the psychological aspects of the FPT program with regard to its ability to support patient's motivational needs, as well as their experience of psychological distress by comparing it to conventional rehabilitation.

## METHODS

The Future Patient Telerehabilitation (FPT) project is a multicenter randomized controlled trial (RCT) (ClinicalTrials.gov: NCT03388918 and the Danish Ethical Committee: N-20160055), focusing on telerehabilitation of patients with HF and carried out from December 2016 to September 2019. The study was conducted in accordance with the Helsinki Declaration, and all participants gave informed consent prior to enrollment in the study.

### PARTICIPANTS

Participants were consecutive patients with HF who had recently been diagnosed with the New York Heart Association (NYHA) class I-IV or acute decompensated HF. The recruitment of patients to the FPT study was based on the following inclusion criteria: (1) Patients diagnosed with HF according to the NYHA class I-IV within the prior two weeks or patients hospitalized with acute decompensated HF within the prior 2 weeks (a maximum of 20% of the included patients were in NYHA class 1), (2) 18 years of age or older, (3) living at home and capable of caring for him/herself, (4) must have basic computer skills, (5) have given informed consent to the current study, and (6) may have a pacemaker.

Patients were excluded from the study if they (a) were pregnant, (b) used psychoactive drugs, such as cannabis, opioids or other drugs, (c) had a previous neurologic, musculoskeletal or cognitive disability, (d) had an active psychiatric history (as noted in the medical record) other than depression or anxiety related to cardiac or other chronic illness, (e) lacked the ability to cooperate (i.e., able to cope with the FPT program), or (f) did not speak Danish.

### PROCEDURE

**Figure 1:**
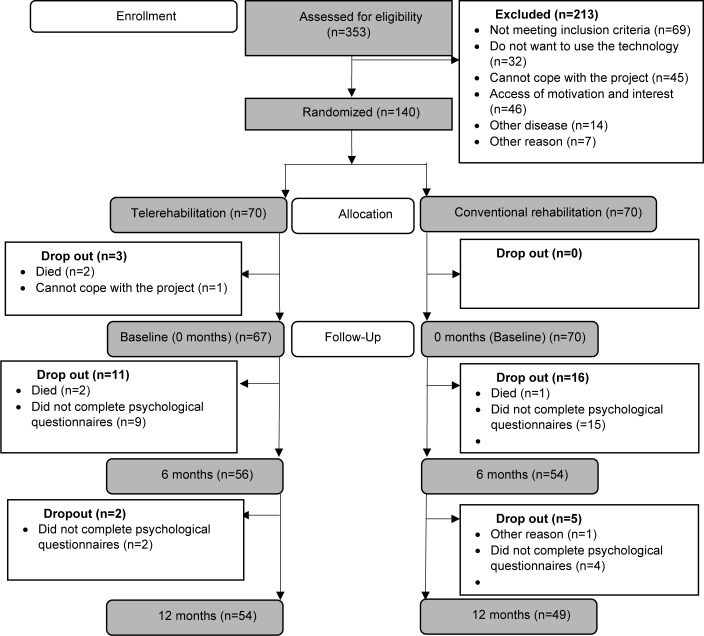
Consort Diagram

Recruitment was conducted through the cardiology departments of four Danish hospitals (Dinesen et al., 2019). A total of 353 patients were assessed for eligibility, of which 140 met the criteria and were enrolled in the FPT project. All eligible included patients were then randomly allocated into two groups (n=70/group). Patients allocated to the conventional rehabilitation (CR) group followed a cardiac rehabilitation program according to the International Cardiac Guidelines, whereas patients allocated to the TR group followed our FPT program. Prior to baseline assessment of psychological measures, there was a loss of three participants in the TR group due to death or lack of ability to cope with the project (see Figure 1). Participants filled in questionnaires for the psychological substudy at baseline (0 months), at the end of phase 2 (6 months), and again at the end of phase 3 (12 months) (see Figure 3).

**Figure 2:**
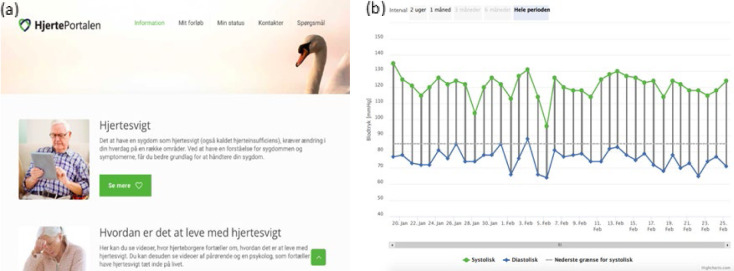
Screen Captures from the Heart Portal

*Note.* (a) Frontpage of the Heart Portal as viewed by the patient, (b) Visualization of the patient's measures of blood pressure

**Figure 3:**
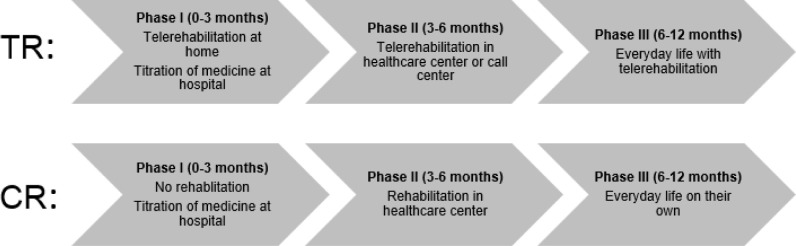
The Three Phases of the Future Patient Telerehabilitation Program

### DESCRIPTION OF THE TELEREHABILITATION INTERVENTION

The FPT program (TR group only) consisted of three phases: (1) titration of medicine at hospital and telerehabilitation at home (0–3 months), (2) participation in the FPT at a health care center or call center (3–6 months), and (3) follow-up with patients carrying out telerehabilitation in everyday life on their own with access to the Heart Portal and their measured values (6–12 months) (see Figure 2). In this way, the FPT provided patients with a coherent and continuous rehabilitation program even when transferring from hospital to community-based rehabilitation. An essential part of the FPT program is access to the Heart Portal, a single access point to a web-based interactive communication platform with multiple purposes (see Figure 2): (a) to provide educational material about HF self-care management, (b) to display current status for the individual patient based on the measures obtained as part of the TR program (i.e., steps, blood pressure, pulse, respiration and weight; see also Figure 2, right), and (c) for communication between health care staff and patients. In combination, the different aspects of the Heart Portal provided the necessary tools and means for communication relevant for the patient to implement and increase self-care. Furthermore, care was taken to design the Heart Portal in line with SDT to support patient motivation. We did not provide any additional psychological intervention in our FPT program but followed the general guidelines for identifying and treating psychological distress in both groups. This allowed us to examine whether the online format resulted in increased psychological distress as suggested by Cranen et al. (2012).

The focus and content of rehabilitation in both groups were similar following general guidelines for HF rehabilitation (Sundhedsstyrelsen, 2015). However, the two groups differed in how they accessed information and communicated with health personnel, as described below. In addition, the TR group-initiated rehabilitation in phase 1 through access to the Heart Portal, whereas the CR group did not start their rehabilitation until phase 2. More details follow.

### THE TR GROUP

The TR group had access to self-monitoring devices/equipment as well as the Heart Portal at home during phases 1–3 (see Figure 3). Phase 1 was carried out from the hospital with the TR coordinator monitoring measured values twice a week, coaching and assisting patients via the Heart Portal. Measured values were automatically transferred to the Heart Portal for visualization (see Figure 2b, right), and patients were encouraged to use these visualizations as feedback on their rehabilitation efforts in addition to using the Heart Portal for collecting information and communicating with the rehabilitation coordinator. Phase 2 resembled phase 1, however, telerehabilitation was now carried out through a health care center or call center, patients digitally transferred data to the platform for visualization, and data was still also monitored by health care professionals. In phase 3, patients took on responsibility for the rehabilitation activities using what they had learned in previous phases and managed their disease on their own with access to the Heart Portal and visualizations of their measured values.

### THE CR GROUP

The participants in the CR group were followed at hospital during phase 1 for titration of medicine, but no rehabilitation was offered (see Figure 3). When HF medicine was stabilized, the group entered a CR program at a health care center in phase 2. They did not receive equipment for monitoring vital signs, nor did they have access to the Heart Portal during phases 1, 2, and 3. In phase 3 the CR group also took on responsibility for their ongoing rehabilitation process.

### MEASURES

#### SOCIODEMOGRAPHIC AND CLINICAL VARIABLES

Sociodemographic variables (age, gender, level of education, work status, civil status) and patients’ experience of their general health were collected using self-report measures, whereas clinical variables (weight, ejection fraction, NYHA class, secondary diagnoses) were extracted from patient's health records.

#### PSYCHOLOGICAL MEASURES

**HealthCare, Self-Determination Theory Packet**. Autonomy, competence, and relatedness were measured using the Health-Care - Self-Determination Theory Packet (HC-SDT), which consists of three questionnaires: (1) the Treatment Self-Regulation Questionnaire (TSRQ), which measures autonomy (15 items); (2) the Perceived Competence Scale (PCS), which measures competence (4 items); and (3) the short version of the Health Care Climate Questionnaire (HCCQ), which measures relatedness (6 items). All items are statements rated on a 7-point Likert scale (from ‘Not at all true’ to ‘Very true’). The TSRQ is divided into three independent subscales: autonomous regulatory style (average of 6 items), controlled regulatory style (average of 6 items) and amotivation (average of 3 items). Scores for the PCS and the HCCQ consist of an average across all items. All statements were rephrased so as to capture the participant's overall experience of autonomy, competence, and relatedness in relation to the rehabilitation process (Williams et al., 2004).

**Hospital Anxiety and Depression Scale^©^.** The Hospital Anxiety and Depression Scale (HADS) is a 14-item self-reporting measure, consisting of two 7-item subscales measuring anxiety and depressive symptoms devoid of somatic symptoms (Zigmond & Snaith, 1983). All responses are indicated on a four-point Likert Scale from 0–3 (score range 0–21). The two subscales have been shown to be internally consistent, as measured by Cronbach's α: HADS-A = .80; HADS-D = .81 (Hermann, 1997). Moreover, a review of 15 studies showed HADS to be a valid and reliable instrument with Cronbach's α for HADS-A ranging from .68–.93 and for HADS-D ranging from .67-.90 (Bjelland et al., 2002). This review also showed ≥8 on both subscales to be an optimal cut-off point as indication of likely psychopathology, with sensitivity and specificity ranging between .70 and .90 for most reviewed studies (Bjelland et al., 2002). Despite controversies in the literature regarding the diagnostic validity of the two subscales of the HADS, we used the original subscales, as we wanted to compare levels of psychological distress across our two intervention arms. Hence, we used the HADS as an indicator of psychological symptoms rather than as a diagnostic tool (Burns et al., 2014; Emons et al., 2012; Martin et al., 2008).

#### DATA ANALYSIS

Prior to analysis, data was screened for missing values and normality of distribution. Missing data was handled according to instructions for the psychological measure in question. If no methods for handling missing items could be found, a mean was imputed when at least 50% of items on the scale in questions were answered (Schafer & Graham, 2002). Patients who dropped out during the study were compared to those who remained using t-test, *χ*^2^ or Fichers Exact test as appropriate.

Baseline comparisons were carried out using t-test, *χ*^2^ or Fichers Exact test as appropriate. To compare our CR and TR group on motivation and psychological distress measures, we first conducted a series of repeated measures ANOVAs (2groupsx3timepoints) using (a) subscales on the HC-SDT, and (b) the HADS. To examine the association between psychological distress and motivation, we repeated these analyses using anxiety and depression as covariates. A significance level of ?=0.05 was adopted for all analyses. Data was analyzed using IBM SPSS Statistics 27.

## RESULTS

### BASELINE CHARACTERISTICS

At baseline there were no significant differences between the TR and the CR group with regard to sociodemographic, clinical, or psychological variables (see Table 1). During the study, a total of 34 patients (24% of the total sample; i.e., 13(38%) in the TR and 21(62%) in the CR group) did not complete the psychological measures or dropped out for other reasons (see Figure 1). The 34 patients who dropped out were generally younger (p<0.01), more likely to be unemployed or on sick leave (p=0.02), and reported more anxiety (p<0.01).

**Table 1:** Baseline Characteristics for the Telerehabilitation and the Conventional Rehabilitation Group

	Telerehabilitation (TR) n=67	Conventional Rehabilitation (CR) n=70	p^†^
Sociodemographic			
Age, mean±SD	61.73±10.75	61.36±11.46	0.84
Male gender, n (%)	51 (49)	54 (51)	0.89
Education, n (%)			0.41
Primary school, unskilled	20 (30)	14 (20)	
Skilled worker, high school	35 (52)	43 (61)	
Bachelor, master, PhD+	13 (19)	12 (17)	
Work status, n (%)			0.28
Unemployed, sick leave	19 (28)	27 (40)	
Employed^‡^	16 (24)	10 (14)	
Retired	32 (48)	33 (47)	
Living with a partner, n (%)	43 (64)	50 (71)	0.46
Clinical parameters			
Weight	85.35±20.35	90.03±20.93	0.19
Systolic blood pressure (mmHg)	124.42±17.67	129.24±18.15	0.10
Diastolic blood pressure (mmHg)	78.97±10.99	81.99±12.20	0.13
Heart rate (beats/min)	78.70±17.76	75.11±16.02	0.22
Ejection fraction (%)	31.80±8.49	32.14±9.38	0.83
NYHA class, n (%)			0.37
I	10 (15)	15 (21)	
II	42 (63)	44 (63)	
III	13 (19)	11 (16)	
IV	2 (3)	0 (0)	
Secondary diagnoses			
Ischemic heart disease, n (%)	3 (4)	4 (6)	0.74
Atrial fibrillation, n (%)	13 (19)	25 (36)	0.04
Claudication, n (%)	0 (0)	1 (1)	1.00
Hypertension, n (%)	9 (13)	18 (26)	0.07
COPD, n (%)	7 (10)	6 (9)	0.71
Diabetes type 1, n (%)	1 (1)	1 (1)	1.00
Diabetes type 2, n (%)	9 (13)	11 (16)	0.71
Kidney disease, n (%)	2 (3)	1 (1)	0.61
Depression, n (%)	1 (1)	3 (4)	0.62

† Analyses were using t-test, Mann-Whitney, *χ*^2^, or Fichers Exact test as appropriate.

‡ Holds any form of employment, no specified hours/week

### MOTIVATION

**Figure 4:**
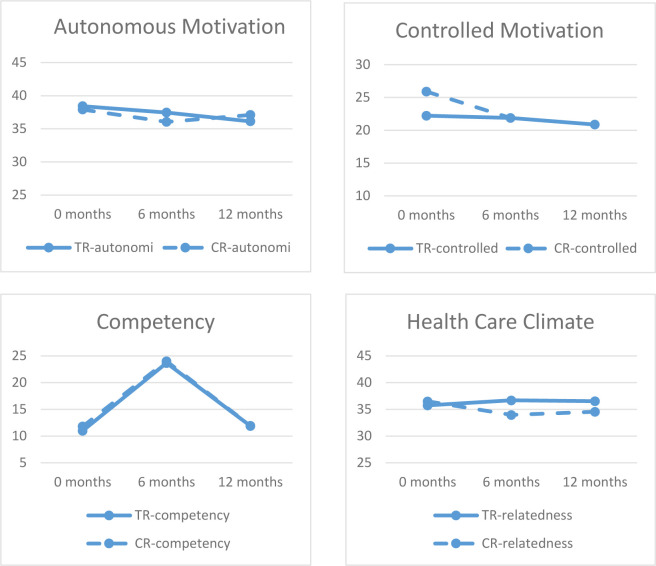
Changes in Motivational Aspects over Time

We examined changes in the various aspects of motivation over the course of the rehabilitation period using ANOVA for repeated measures (group_TR vs CR_ x time_3_). We found no significant differences over time except for competence (F_1.59_=823.59, p<0.00), where both groups showed an initial increase during phases 1 and 2, although this increase dropped to the initial level after phase 3 (see Table 2). With respect to controlled motivation, we found an initial difference between groups, with our CR group reporting higher levels of controlled motivation compared to our TR group (see Figure 4b). This difference was consistent throughout the study period (F=6.45, p=0.01). With regard to relatedness, a significant interaction was observed, with the CR group initially reporting slightly higher levels of relatedness. However, their scores dropped significantly over time and fell to a level below that of the TR group (F_2_=3.41, p=0.04) (see Figure 4).

**Table 2:** Results of Unadjusted Analyses for All Outcome Measures over Time

Time point	0 mos. Mean(SD)	6 mos. Mean(SD)	12 mos. Mean(SD)	F Time Group Interaction	p
Motivation					
Autonomous Motivation				F_1.93_=2.76	**0.07**
TR_(n=51)_	38.41(4.01)	37.44(6.20)	36.12(8.20)	F=0.11	0.74
CR_(n=48)_	37.90(5.43)	36.04(7.32)	37.10(5.60)	F_1.93_=1.36	0.26
Controlled Motivation				F_2_=.95	0.39
TR_(n=51)_	22.22(8.86)	21.88(9.04)	20.87(9.16)	F=6.45	**0.01**
CR_(n=48)_	25.90(8.21)	25.56(8.54)	25.10(9.27)	F_2_=.08	0.92
Competence				F_1.59_=823.55	**0.00**
TR_(n=50)_	11.00(2.38)	23.68(3.76)	11.90(1.96)	F=.68	0.41
CR_(n=48)_	11.81(2.09)	24.00(4.33)	11.95(2.09)	F_1.59_=.45	0.59
Relatedness				F_2_=.59	0.56
TR_(n=50)_	35.75(6.31)	36.70(4.76)	36.54(6.35)	F=1.70	0.20
CR_(n=47)_	36.51(6.43)	33.97(7.55)	34.55(6.73)	F_2_=3.41	0.04
Psychological distress					
Anxiety				F_1.77_=14.19	**0.00**
TR_(n=51)_	5.96(3.87)	4.49(3.83)	4.66(4.38)	F=.96	0.33
CR_(n=47)_	5.43(3.56)	3.67(3.77)	3.98(3.82)	F_1.77_=.09	0.89
Depression				F_1.48_=3.03	0.07
TR_(n=51)_	4.29(3.92)	3.93(4.02)	4.29(4.17)	F=2.35	0.13
CR_(n=47)_	3.77(3.09)	2.70(3.51)	2.94(3.35)	F_1.48_=1.24	0.29

### PSYCHOLOGICAL DISTRESS

**Figure 5:**
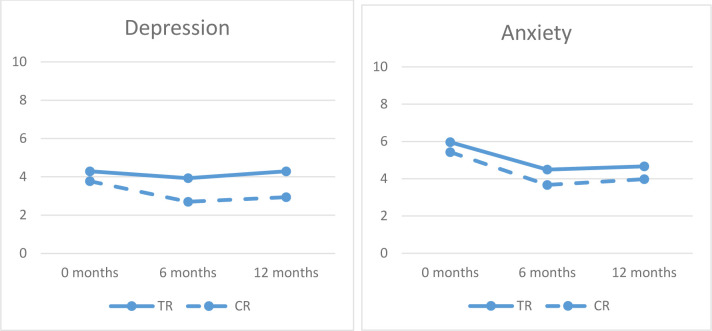
Changes in Psychological Distress over Time

For both groups, we found significant decreases in anxiety over time (F_1.77_=14.19, p<0.00), whereas changes in depression scores were non-significant (F_1.48_=3.03, P = 0.07). Mean scores for both anxiety and depression indicate an initial drop in symptom level, most pronounced for anxiety. However, mean scores on both scales increase slightly again from 6–12 months (see Table 2 and Figure 5).

### EXAMINING THE ROLE OF PSYCHOLOGICAL DISTRESS IN MOTIVATION

As psychological distress may influence motivation, we adjusted the above analyses of motivational aspects for baseline levels of anxiety and depression to address this issue in our sample. In line with our unadjusted analyses, we found that competence significantly changed over time (F_1.62,_=232.92, p<0.00). In addition, depression was associated with a decreased experience of competence (F_2_=3.41, p=4.04). The time x group interaction observed in relation to relatedness also remained significant when adjusting for depression and anxiety (F_2_=3.59, p=0.03). In addition, a main effect of depression on relatedness (F=5.46, p=0.02) and an interaction between time and depression were also observed _on_(F_2_=3.21, p=0.04) with depression generally being associated with an impaired experience of relatedness.

## DISCUSSION

The FPT project aimed to develop a TR program that motivated patients to engage in disease management through TR and maintain this motivation after the formal TR program was completed. Overall, our results indicate that TR motivates patients to at least the same degree and on some aspects of motivation more successfully than CR. We also examined whether telerehabilitation was associated with increased psychological distress, and whether psychological distress was associated with motivation. We found that both TR and CR were associated with reductions in symptoms of anxiety as a measure of psychological distress. Importantly, we found no differences in psychological distress between the two groups. Our results also indicate a significant association between depression and aspects of motivation independent of the mode of rehabilitation delivery.

We evaluated our primary aim by investigating aspects of motivation as outlined in the SDT framework, that is, autonomy, competence, and relatedness. The degree of autonomy in our two groups was examined using two specific aspects of motivation, (a) controlled (extrinsic) and (b) autonomous (intrinsic) motivation. Overall, we found that our CR group expressed a higher level of controlled motivation compared to our TR group, whereas the two groups reported comparable levels of autonomous motivation (see Table 2 and Figures 4a and 4b). This suggests that the CR group were more likely to be dependent on external sources of motivation, such as health personnel, compared to our TR group, both during and following completion of a rehabilitation program after HF. As such, it seems that our TR program was superior in encouraging patients to depend on intrinsic rather than extrinsic motivation. According to the SDT, intrinsic motivation may in turn be reflected in more sustained motivation for rehabilitation and disease management long-term. Combined with our previous findings (Spindler et al., 2019), these results support that TR may be equally and maybe even more motivating over time compared to CR.

Developing patients’ competence in disease management is an essential part of the rehabilitation process. In the FPT program, patient competency was developed using a number of digital tools. The HeartPortal provided patients with information about their disease, ongoing feedback about current health status, and digital dialogue with health personnel. Over time, our participants reported identical patterns of competence across groups with an initial increase from baseline to the end of phase 2, which declined again during phase 3. This latter finding was surprising and in contrast to our previous findings (Spindler et al., 2019), although, Rahman and colleagues (2015) found similar results with regard to competence in relation to physical exercise as part of CR. It is unlikely that competencies obtained in phase 1 and 2 were forgotten during phase 3. However, it may reflect that patients felt more confident regarding their own competency during phases 1 and 2, compared to phase 3 when they managed their disease independently.

The final aspect of motivation according to SDT is relatedness. Our results indicate that our CR group experienced less relatedness over time compared to our TR group, that is, the digital relatedness in the TR group was superior to the in-person relatedness experienced by the CR group. In addition, the TR group maintained this experience of digital relatedness also when they were completing the rehabilitation activities independently in phase 3. This contrasted with our previous findings (Spindler et al., 2019) showing no differences in relatedness across groups. The differences identified here may reflect that the HeartPortal contained additional features for communication between patients and health care personnel compared to the Active Heart website used in the Teledi@log project (Spindler et al., 2019) – a precursor to the FPT project (Joensson et al., 2019). These additional features were developed as part of the initial participatory design process based on expressed needs and desires by patients, partners, and health care staff.

Building on a recent meta-analysis linking specific techniques and interventions to the various aspects of motivation (Gillison et al., 2019), we created a practically relevant description of our findings. The FPT program seems to support the patients’ feeling of autonomy during rehabilitation on par with CR, whereas CR invited slightly more controlled motivation compared to TR. In addition, the FPT created a supportive health care climate fostering relatedness, which in turn may be superior to the CR program in inviting patients to engage in rehabilitation tasks. Taken together our results support that telerehabilitation is a viable alternative or supplement to conventional rehabilitation, as it supports patient motivation. As such, our results are in line with the general consensus in the literature stating that TR is a well-accepted, safe, and effective alternative or supplement to CR (Anderson et al., 2017; Brouwers et al., 2020; Frederix et al., 2015; Piotrowicz, 2017; Spindler et al., 2019).

We also examined whether TR was associated with increased psychological distress. This was not the case. Instead, we found that regardless of rehabilitation group, anxiety levels decreased and that changes in levels of depression did not reach statistical significance. These differential patterns of change in specific symptoms of psychological distress may be explained by looking at how anxiety and depression are conceptualized in acceptance and commitment therapy (ACT) (Batten, 2011). In turn, this may also illustrate how patients’ experience their disease over time. In ACT, anxiety is considered associated with experiential avoidance of things that may evoke feelings of anxiety (Batten, 2011), e.g., being confronted with having heart failure. To address experiential avoidance, ACT interventions focus on making patients divert from experiential avoidance (Batten, 2011), which for patient with HF may be described as being exposed to the reality of their disease as HF rehabilitation may serve as a form of exposure in line with ACT exposure-based interventions (Batten, 2011). As patients are exposed to everyday life with HF and engage in monitoring symptoms as part of disease management, these activities may result in a reduction in HF-related anxiety. In addition, learning to accurately interpret symptoms by engaging in disease management and rehabilitation may also provide relevant exposure to living with HF that contributes to decrease in HF-related anxiety that patients’ experience. In essence, HF rehabilitation combined with natural remission, may be why we see this decrease in anxiety symptoms across both groups. Of note, our analysis is based on group means, hence identifying those who do not experience remitting anxiety over time is still a priority to increase the outcome of rehabilitation efforts.

In contrast, ACT conceptualizes depression as reflecting an individual's way of escaping from difficult thoughts, feelings, and memories (Batten, 2011). Depression is also considered to be associated with negative cognitions about previous experiences and the self (Batten, 2011). As argued above, the future-orientation in fear or anxiety associated with HF may be, to some extent, addressed by the exposure to HF during rehabilitation However, the same may not hold for the retrospective focus in depression (Batten, 2011). Disease management and rehabilitation do not provide immediate exposure for negative cognitions about previous experiences or the self, nor do they require that the patient is exposed to difficult thought, feelings, or memories to perform self-care.

Lastly, when examining the role of psychological distress in relation to motivation, our initial findings regarding motivation were confirmed. In addition, the adjusted analysis indicated that depression was independently associated with both decreased competency and a decreased experience of relatedness. These findings underscore the detrimental role of psychological distress, and especially that of depression in relation to motivation. To our knowledge, this association has not been examined in HF previously, although a study in patients with diabetes showed a similar association between depression and motivation (Egede & Osborn, 2010). In sum, these findings underline the importance of addressing psychological distress as part of TR, not just during the program, but also prior to engaging patients in rehabilitation programs. Patients who are dealing with psychological distress may not have the necessary surplus resources to engage in rehabilitation (Pâquet et al., 2005). This may have been what we found in our drop-out analysis which showed that psychological distress was associated with higher risk of dropout. This suggests that patients who experience psychological distress may not be as willing to partake in research studies and perhaps also in rehabilitation programs. Identifying and intervening in relation to psychological distress, especially depression, may improve the patient's mental health and thereby increase adherence to rehabilitation, as well as provide the possibility for a fuller life with HF, less hampered by psychological distress.

Taken together, the current results support the use of TR as a viable substitute for CR, which may be especially relevant during the ongoing COVID-19 pandemic. Few studies have directly assessed motivational processes in cardiac rehabilitation (Rahman et al., 2015; Sweet et al., 2011), and, to our knowledge, our current and previous studies (Spindler et al., 2019), are the only studies examining motivational aspects of telerehabilitation programs. Interestingly, we found that TR provided more relatedness compared to CR, which is surprising given concerns that TR is associated with less contact with health care professionals (Cranen et al., 2012; McLean et al., 2011). As such, our results indicate that relatedness is not contingent upon physical contact to health care personnel, and that digital relatedness is experienced as being on par with real-life relatedness, as our TR group reported experiencing more relatedness compared to our CR group. This is a unique finding of the FTP study. Given that experiencing relatedness is considered an essential for motivation we believe that this should be addressed further in future studies.

### STUDY LIMITATIONS

Our results should be considered with the following limitations in mind. Although our dropout rates were of an expected magnitude, we nevertheless experienced a skewness in our dropout-rates, with more patients dropping out of our CR group, and with dropout being associated with increased psychological distress. This suggests that those patients experiencing the highest levels of psychological distress did not participate over time, regardless of the rehabilitation format. This may have had some bearing on our results regarding psychological distress, and perhaps our other outcome measures. That is, our results may only pertain to patients with HF with psychological distress that does not interfere with their participation in the study. In addition, these findings may also suggest that psychological distress *per se* is associated with decreased adherence, as mentioned in the discussion, and that alleviating psychological distress may be a prerequisite for successful rehabilitation. In addition to being part of the CR group, younger age, and being unemployed or on sick leave were also associated with higher risk of dropout. It is unclear what these observations reflect, but perhaps the lack of contact with health personnel in the CR group may make participation in the study less favorable. With regard to younger age, these persons may not find themselves in need of rehabilitation, and hence decline further participation. In contrast, those unemployed and on sick leave may find dealing with these issues draining their resources as described in relation to psychological distress. However, some of the factors associated with dropout co-occurred in patients and our current study cannot provide any specific explanation for these findings. Future research may be able to elucidate whether these factors are important for retaining patients in rehabilitation and even research studies. For those patients retained in the study, we made an extra effort to ensure that there was a limited number of missed items on questionnaires (by several reminders), hence, our data quality for patients retained was good, although, we still had some missing values. These were handled as described in the Methods section.

In addition, we did not specify a hypothesis stating that we wanted to examine whether TR was superior to CR, but that we wanted to evaluate motivation and psychological distress across our two groups. As such, our non-significant results are therefore not negative *per se*, but they cannot readily be assumed to infer non-inferiority, as our study was not powered sufficiently to support such an analysis (Schumi & Witter, 2011). Although a power analysis had been carried out for the main study, this was aimed at identifying significant changes on the KCCQ aiming for superiority testing (see Dinesen et al., 2019), rather than non-inferiority testing. In sum, future studies are needed to address the issue of non-inferiority between telerehabilitation and conventional rehabilitation.

## CONCLUSION

The Future Patient Telerehabilitation (FPT) project used participatory design to develop a telerehabilitation program for patients with HF on par with conventional rehabilitation while also providing support for patient motivation for self-care. Our results indicate that the FPT program was comparable to conventional rehabilitation with regard to motivating patients. Patients in the telerehabilitation group reported a significantly better experience of digital relatedness compared to our conventional rehabilitation group. In addition, we found no differences between groups with regard to psychological distress, although our results indicate that depression in general is associated with decreased motivation. Taken together, our results support that telerehabilitation is a viable alternative or supplement to conventional rehabilitation, as it supports patient motivation and is not associated with increased psychological distress.
